# Management of Blood Pressure During and After Recanalization Therapy for Acute Ischemic Stroke

**DOI:** 10.3389/fneur.2019.00138

**Published:** 2019-02-21

**Authors:** Jeffrey R. Vitt, Michael Trillanes, J. Claude Hemphill

**Affiliations:** ^1^Department of Neurology, University of California, San Francisco, San Francisco, CA, United States; ^2^Department of Pharmaceutical Services, University of California, San Francisco, San Francisco, CA, United States

**Keywords:** acute ischemic stroke, cerebral autoregulation, hypertension, ischemic penumbra, embolectomy

## Abstract

Ischemic stroke is a common neurologic condition and can lead to significant long term disability and death. Observational studies have demonstrated worse outcomes in patients presenting with the extremes of blood pressure as well as with hemodynamic variability. Despite these associations, optimal hemodynamic management in the immediate period of ischemic stroke remains an unresolved issue, particularly in the modern era of revascularization therapies. While guidelines exist for BP thresholds during and after thrombolytic therapy, there is substantially less data to guide management during mechanical thrombectomy. Ideal blood pressure targets after attempted recanalization depend both on the degree of reperfusion achieved as well as the extent of infarction present. Following complete reperfusion, lower blood pressure targets may be warranted to prevent reperfusion injury and promote penumbra recovery however prospective clinical trials addressing this issue are warranted.

## Introduction

Stroke is a common neurologic emergency worldwide with an overall growing incidence particularly in low to middle income countries where there has been over a 100% increase in stroke events over the past four decades (1). Approximately 85% are ischemic in origin and for the past two decades, intravenous tissue plasminogen activator (IV t-PA) has been the mainstay of treatment for patients with acute ischemic stroke (AIS) presenting within 3, and then expanded to 4.5, hours since last known well (2, 3). IV t-PA reduces the rate of functional dependence in up to one-third of individuals, but many AIS patients do not benefit from this treatment (2, 3). Over the past several years, multiple landmark studies have provided overwhelming evidence that intraarterial therapy (IAT) with mechanical thrombectomy, performed within 6 h of last known well in large vessel occlusion (LVO), leads to significantly improved functional outcomes and reduced mortality (4–7). More recently, the DEFUSE III and DAWN trials demonstrated that IAT can benefit patients treated out to 16 and 24 h if they have a favorable mismatch pattern on perfusion imaging (8, 9). While these breakthroughs have altered the paradigm of acute stroke management and can be considered as part of routine care, several unresolved issues remain regarding the optimal treatment of patients presenting with AIS, particularly regarding hemodynamic management. Though blood pressure (BP) elevation is common in AIS, the prognostic significance of this is unclear (10, 11). Some studies have found a correlation between hypertension and poor outcomes while others have reported inverse relationships (12–15). Furthermore, the guidelines for hemodynamic treatment following thrombolytic therapy in AIS are largely extrapolated from the IV t-PA trials as well as retrospective analyses (16). Thus, high quality evidence to guide management after IAT is lacking. The purpose of this review is to discuss the physiology and available data regarding hemodynamics in AIS with particular focus on how blood pressure might be optimally managed throughout the revascularization process.

## Pathophysiology

In order to understand the principles of blood pressure management during AIS revascularization, it is useful to review fundamental aspects of blood flow in cerebral ischemia and infarction. Following complete cessation of cerebral blood flow (CBF) there is loss of normal neuronal electrical activity within seconds due to energy failure, disruption of ion homeostasis and membrane depolarization (17, 18). If perfusion is not restored within minutes, irreversible injury ensues leading to infarction (19). Ischemic stroke, however, is a focal process and there is rarely complete loss of CBF. Instead, surrounding the occluded vascular territory exist areas of mild hypoperfusion with intact function, ischemic tissue which remains salvageable but with dysregulated cellular processes (termed penumbra), and infarction with irreversible damage (17).

Early clinical studies using carotid clamping revealed that the risk of transitioning from ischemic to infarcted tissue depends on both the magnitude and duration of hypoperfusion (17). Furthermore, if CBF is reestablished in a timely manner the ischemic tissue may be salvaged and restored to normal function. Neurons within the penumbra are highly vulnerable to changes in local perfusion pressure, either from edema, alterations in systemic BP, or changes in cerebral vasoreactivity, and maintain a relatively preserved oxygen consumption despite lower CBF due to an increase in the oxygen extraction fraction (20, 21). While the infarction threshold largely depends on the duration and extent of hypoperfusion, individual factors including vascular compliance and collateral vessels between both intracranial and extracranial circulations can influence the resilience of the penumbra (17). Following acute vessel occlusion, the perfusion pressure distal to the clot falls leading to a pressure gradient in which retrograde flow commences through collaterals thereby achieving a sufficient level of CBF to maintain penumbra viability (22). In this setting, drops in BP or increases in tissue pressure from local mass effect can lead to an attenuation of the collateral gradient and exacerbate ischemia (23). This is demonstrated clinically in AIS as patients with more robust collaterals often have a lower BP, likely from adequate perfusion to the penumbra, and improved clinical outcomes relative to those with poor collateralization (24, 25). Furthermore, the presence of a robust collateral circulation predicts a higher likelihood of recanalization following IAT and, in cases where the procedure is unsuccessful, there is a reduced infarct volume compared to patients with poor collateralization (26, 27).

In the healthy brain, CBF is tightly regulated to meet regional metabolic demand and this is accomplished through the process of cerebral autoregulation whereby resistance-level blood vessels constrict or dilate across a range of systemic pressures (typically a mean arterial pressure [MAP] between 50 and 150 mmHg) in order to maintain a more constant flow (28). When pressures fall below the lower limit of autoregulation, surrounding brain parenchyma becomes ischemic and eventually infarcted unless CBF is rapidly restored. Conversely, when MAP rises above the capacity of cerebral autoregulation, a linear increase in CBF occurs leading to edema and hemorrhage. In AIS, the disruption of blood flow results in dysregulation of multiple cellular processes which may include autoregulatory mechanisms within the penumbra, thus making CBF directly dependent on systemic pressures (28, 29). Though early studies using radiotracer injection confirmed changes in CBF within the ischemic hemisphere in proportion to alterations in MAP, the resolution of these techniques did not allow for differentiation of penumbra from core infarct (28). Newer studies have had various findings, with reports of impaired autoregulation both globally and within the ischemic hemisphere contrasting with a recent study that found no change in regional CBF following alterations in MAP using high resolution positron emission tomography (PET) (30, 31). While these conclusions were derived from measures of static autoregulation, in which regional changes in CBF were assessed at a single time point after BP manipulation, recent focus has shifted toward dynamic autoregulation using techniques such as transcranial doppler to track instantaneous changes in blood flow in response to BP fluctuations (28, 30). In contrast to static autoregulation, which is often preserved in AIS, recent studies have revealed that dynamic autoregulation may be particularly vulnerable to ischemic insult and can remain abnormal for several weeks after presentation (30, 32). Though impairments in dynamic autoregulation appear common across a spectrum of stroke subtypes and may indicate selective damage to central autonomic control networks, the clinical relevance remains unclear and is the subject of ongoing investigation (30, 33).

While reestablishing CBF is essential for survival of ischemic tissue, reperfusion itself can contribute to significant neurologic injury in the form of infarction, edema and hemorrhagic transformation (34, 35). Reperfusion injury is a complex and incompletely understood process however several important underlying pathophysiologic mechanisms have been identified. Immediately following recanalization there often is a dramatic increase in CBF, likely as a result of impaired autoregulation as well as release of vasodilatory substances, which leads to hyperperfusion and the potential for secondary cellular injury (18, 36). The magnitude of cerebral hyperemia seems to be influenced in part by the duration of ischemia and in MRI studies, hyperperfusion following thrombolysis was most commonly observed in areas of pretreatment hypoperfusion and was an independent predictor of eventual infarction (36). After recanalization there can also exist a paradoxical hypoperfusion state, termed no-reflow phenomenon, which can lead to permanent infarction and is thought to result from microvascular dysfunction related to astrocyte and endothelial cell swelling as well as increased inflammation and platelet aggregation (37, 38). On a cellular level, reperfusion after prolonged ischemia leads to mitochondrial overproduction of toxic reactive oxygen species causing inflammation and triggering the release of extracellular matrix metalloproteinases (MMP) which enzymatically degrade the endothelial basal lamina and increase microvascular permeability (39, 40). Loss of blood brain barrier (BBB) integrity in turn leads to vasogenic cerebral edema formation and in clinical studies is a strong predictor for hemorrhagic transformation and poor neurologic outcome following revascularization (41).

## Blood Pressure in Acute Stroke

Elevated BP is common in patients presenting with AIS, with one study involving more than 250,000 patients demonstrating a systolic blood pressure (SBP) > 140 mmHg in approximately three-fourths of patients (10). Severe hypertension is also relatively common with nearly 10% of patients presenting with SBP > 200 mmHg (42). Multiple observational studies have identified elevated BP as a risk factor for cerebral edema, hemorrhage and generally worse clinical outcomes following AIS (43–45). However, this association does not necessarily indicate a causative relationship. Instead, hypertension may be a marker of stroke severity, such as in the case of carotid terminus occlusion or poor collateralization, where spontaneously elevated blood pressure may serve as a compensatory mechanism to maintain cerebral perfusion (25, 46). Under which circumstances these mechanisms become maladaptive and contribute directly to cerebral injury remains uncertain and requires further clarification through clinical trials.

In several cohorts, a U-shaped relationship exists between BP and outcome in AIS in which both extremes of BP have prognostic significance for death and disability. In a retrospective analysis of the International Stroke Trial, patients presenting with SBP 140–179 mmHg had the lowest likelihood of death or dependency at 6 months with a nadir at around 150 mmHg (42). For every 10 mmHg above a SBP of 150 mmHg, patients had a 3.6% increase in the risk of death and a 4.2% increased risk of recurrent stroke within the next 6 months. For patients with a SBP > 200 mmHg there was more than a 50% increase in the risk of stroke. Conversely, relative hypotension was also detrimental with a 17.9% increased risk of death for every 10 mmHg drop below 150 mmHg; patients with SBP < 120 mmHg had the worst outcomes and a higher incidence of coronary events. Similar findings have been reported in other studies with slightly different BP thresholds conferring the most favorable outcomes. In work done by Vemmos and colleagues, the best outcomes were observed with SBP values around 130 mmHg while in a study from the Mayo Clinic, the optimal threshold for SBP seemed to be in the range of 156–220 mmHg with a nearly two-fold increase in risk of mortality with episodes of hypotension (47). Similarly, in work done by Castillo et al. patients had lower mortality and more functional independence when presenting with a SBP near 180 mmHg, with final infarct volumes being highest among patients with SBP well above or below this value (15). Interestingly, abrupt declines in SBP (>20 mmHg) were identified as the strongest predictor of poor outcome and were associated with a larger final infarct volume of over 60 ml, suggesting that dynamic BP changes may be particularly injurious to vulnerable ischemic tissue.

In clinical studies, dynamic fluctuations in BP have been identified as a strong prognostic marker in AIS and increase the risk of intracranial hemorrhage following IV t-PA (12). BP variability may be particularly harmful in the setting of large territory infarcts, where it has been independently linked to worse clinical outcomes (12, 48). In one study, patients with BP variability seemed to have worse outcomes in the presence of robust collaterals despite otherwise similar hemodynamic profiles (48). The reasons behind these findings are not entirely clear, however it may be related to increased transmission of fluctuating pressures to the ischemic penumbra. While the impact of BP variability seems to be more apparent in the initial stages of ischemia, one study found day-to-day variability over the course of 1 week was higher in patients with poor outcomes at 1 year (14). Overall, it appears that ischemic tissue may be particularly susceptible to fluctuations in systemic BP, likely as a result of impaired autoregulation and narrow ischemic thresholds, leading to either hypoperfusion with infarction or surges in perfusion with resulting edema (15, 28). Table 1 summarizes results from several observational studies related to blood pressure and ischemic stroke outcome.

**Table 1:** Observational studies examining impact of blood pressure in acute ischemic stroke.

**References**	**Patients**	**Observed optimal blood pressure**	**Main findings**
Leonard-Bee et al. (42)	17,398 patients with AIS enrolled in IST	SBP 140–179 mmHg	U-shaped relationship between baseline SBP and outcomes such that for every 10 mmHg below 150 mmHg there was an increase in early death by 17.9% and death or disability at 6 months of 3.6%. For every 10 mmHg above 150 mmHg there was a 3.8% increase in risk of early death. Low SBP independently associated with fatal coronary events.
Castillo et al. (15)	304 patients with hemispheric AIS	BP 180/100mmHg	U-shaped association with increase in poor outcome by 25% for every 10 mmHg below SBP 180 mmHg and 40% for every 10 mmHg below SBP 180 mmHg. Decrease in SBP > 20 mmHg associated with highest final infarct volumes.
Vemmos et al. (49)	1121 patients admitted for AIS or ICH and enrolled in “Athens Stroke Registry”	BP 121–140/81–90 mmHg	U-shaped relationship with 40% mortality for SBP < 101 mmHg and 46.7% for SBP >220 mmHg. Mortality 45.8% for DBP < 61 mmHg and 50% for DBP>120 mmHg. Low admission SBP associated with heart failure and coronary heart disease while high SBP was associated with lacunar stroke and history of HTN.
Stead et al. (47)	357 patients presenting to ED with AIS	BP 155–220/70–105 mmHg	U-shaped associated with worse outcomes noted for DBP < 70 mmHg or >105 mmHg and for SBP < 155 mmHg and >220 mmHg. MAP 100–140 mmHg was associated with the most favorable outcomes.
Ishitsuka et al. (43)	1,874 patients with first ever AIS	BP < 165/90 mmHg	Linear relationship between post-stroke BP and outcomes such that higher BP was associated with higher risk of neurologic deterioration and poor functional outcomes.

*AIS, acute ischemic stroke; BP, blood pressure; SBP, systolic blood pressure; IST, International Stroke Trial; DBP, diastolic blood pressure; ICH, intracerebral hemorrhage; HTN, hypertension; ECASS, European cooperative acute stroke study*.

## Acute Ischemic Stroke Hemodynamic Management

### Current Practice Guidelines and Stages of Management

In the 2018 guidelines for management of AIS from the American Heart Association (AHA), BP may be permitted up to 220/120 mmHg in patients presenting with AIS who are not candidates for either IV t-PA or IAT and do not have another contraindication to an elevated BP (16). In patients where such a contraindication exists, such as an acute coronary event, decompensated heart failure or preeclampsia, lowering the BP should be individualized, with an initial decrease by 15% recommended. For patients who are eligible for IV t-PA therapy, it is recommended that the BP be maintained below 185/110 mmHg during the infusion and 180/105 mmHg for the following 24 h. These thresholds are largely extrapolated from thrombolysis trials in myocardial infarction as well as pilot data prior to the National Institute of Neurologic Disorders (NINDS) t-PA Trial and have subsequently been validated in retrospective studies where higher BP significantly increased the risk of hemorrhagic transformation (2, 16, 45, 50). In contrast, there is a paucity of prospective data to help guide the management of BP in IAT, particularly when considering the extent of post-procedural recanalization. Aside from the ESCAPE trial, all the pivotal thrombectomy trials used a BP cutoff of 185/110 mmHg, as patients were also potential candidates for IV t-PA, making it challenging to extrapolate the impact of different hemodynamic targets (4–7). As such, the current AHA guidelines recommend maintaining the BP below 185/110 mmHg, but acknowledge the lack of randomized controlled trials to substantiate this position (Class IIb recommendation) (16).

**Figure 1:**
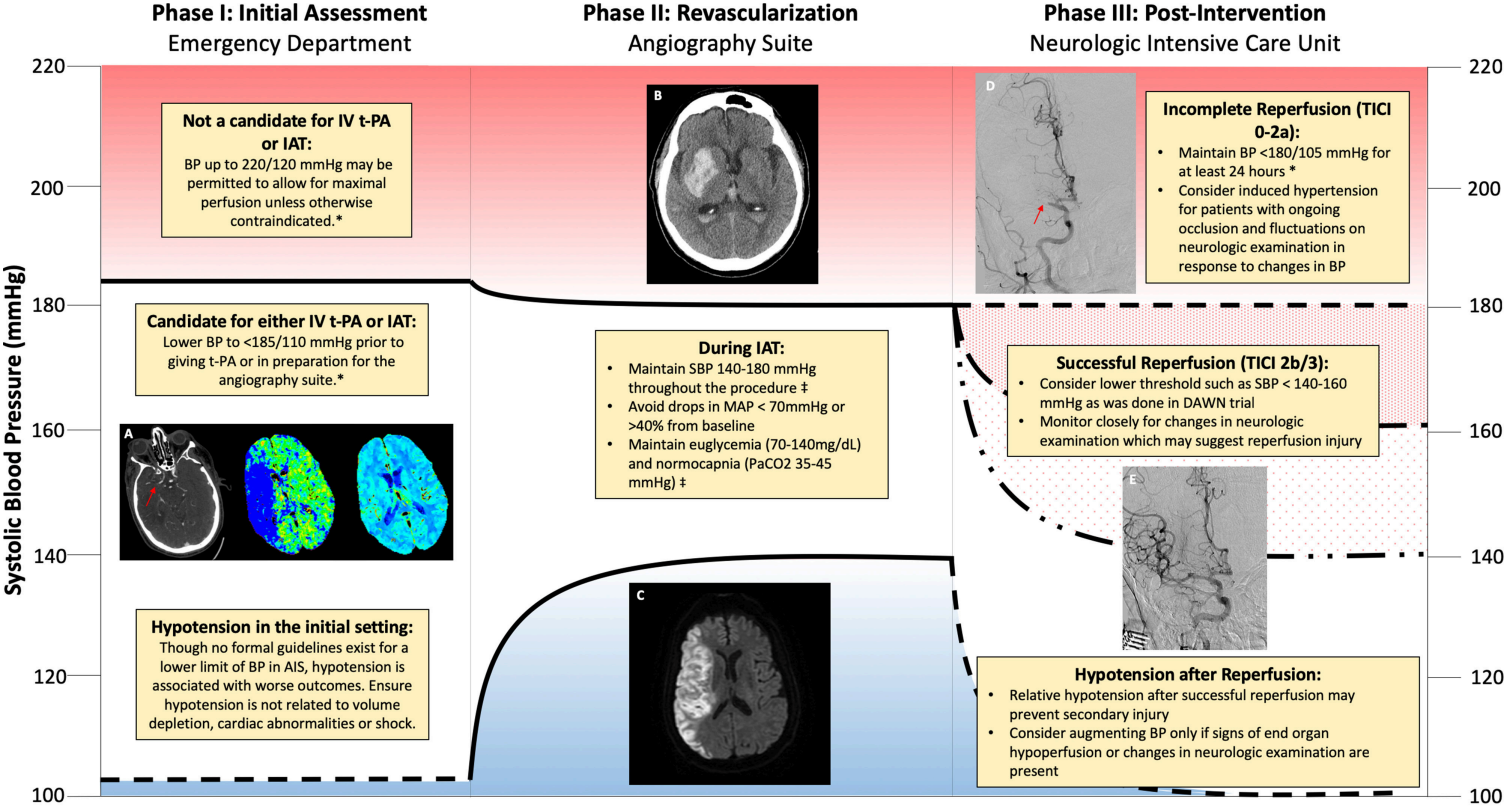
Schematic of hemodynamic management during different phases of revascularization for acute ischemic stroke. **(A)** Computerized tomography (CT) angiogram demonstrating non-opacification (red arrow) of the right middle cerebral artery (R MCA) consistent with large vessel occlusion. Mean transit time increased within the territory of the R MCA with preserved cerebral blood volume consistent with penumbra. **(B)** CT revealing hemorrhagic transformation within the R MCA territory. **(C)** Magnetic Resonance Imaging (MRI) demonstrating increased signal on Diffusion Weighted Imaging (DWI) within the R MCA territory consistent with an acute infarction. **(D)** Absent reperfusion (red arrow) within the R MCA (Thrombolysis in Cerebral Infarction (TICI) Score of 0). **(E)** Complete Reperfusion within the R MCA (TICI Score of 3). *American Heart Association/American Stroke Association 2018 Guidelines for the Early Management of Patients with Acute Ischemic Stroke. ^‡^Society for Neuroscience in Anesthesiology and Critical Care Expert Consensus Statement: Anesthetic Management of Endovascular Treatment for Acute Stroke.

The overall approach to AIS treatment is multifaceted and relies upon optimized systems of care to identify, triage and treat patients presenting with acute neurologic symptoms with the goal of reestablishing cerebral perfusion and minimizing secondary injury. In order to appreciate aspects of hemodynamic management in AIS, it is constructive to separate it into stages of treatment beginning with the initial assessment, during revascularization, and post-intervention (see Figure 1). Each stage presents slightly different considerations regarding hemodynamic management as well as physiologic optimization strategies aimed at maximizing the chances of good recovery.

### Phase I: Initial Assessment

Hypertension is very common in AIS with 21–50% of patients presenting with blood pressure higher than the threshold eligible to receive IV t-PA (11, 42, 49). The largest concern regarding t-PA therapy is the risk of hemorrhage which was approximately 6% in the initial NINDS t-PA trial and has largely been validated in subsequent analysis when adhering to the same protocol BP thresholds (2, 51, 52). An analysis of the SITS-ISTR (Retrospective Analysis From Safe Implementation of Thrombolysis in Stroke-International Stroke Thrombolysis Register) trial demonstrated a linear relationship between SBP and symptomatic hemorrhage with the risk being four times higher for patients with a SBP >170 mmHg compared to those at 141–150 mmHg (53). In light of this data it is prudent to achieve the 185/110 mmHg threshold for patients who are deemed eligible for IV t-PA therapy. However, caution must be applied to avoid significant fluctuations in blood pressure which may be associated with adverse events. In the NINDS t-PA trial, patients treated with BP-lowering agents had more abrupt declines in BP and worse outcomes at 3 months compared to hypertensive patients not treated with medications (54). In subsequent analysis, an overall decline in SBP > 50 mmHg or an acute drop of > 30 mmHg was associated with poor functional outcomes while an acute drop of > 60 mmHg increased the risk of death by 2-fold (55).

For patients who are candidates for IAT there is considerably less data to guide management of initial blood pressure. However, several insights are apparent through retrospective analyses. In the Mechanical Embolus Removal in Cerebral Ischemia (MERCI) trial, patients presenting with SBP > 150 mmHg were less likely to achieve recanalization compared to those with lower pressures despite similar thrombus characteristics (56). Since higher BP is often associated with poor collaterals, these findings may signify that these patients have a maximal pressure gradient against the clot leading to impaction and more challenging retrieval (25). The impact of elevated SBP in IAT may not be related solely to revascularization success, however, as one cohort study found that lower admission SBP was independently associated with more favorable 3 month outcome even after statistical adjustment for complete revascularization (57). In the Multicenter Randomized Clinical Trial of Endovascular Treatment of Acute Ischemic Stroke in the Netherlands (MR CLEAN) trial, a U-shaped relationship was apparent between SBP and poor functional outcome, with a nadir (most favorable BP) of 120 mmHg, and a 21% increase in the relative risk of hemorrhage for every 10 mmHg above this value (58). It should be noted that there was no interaction between BP and the benefit of IAT on clinical or radiographic measures, thus indicating that thrombectomy is safe and effective across a range of BP. Similar results were noted in the Endovascular Treatment in Ischemic Stroke registry, where there was a 3.78 and 1.81 times higher risk of mortality for SBP < 110 mmHg and >180 mmHg respectively compared with 150 mmHg (59). Similar to other studies in AIS, the extremes of BP appear to have negative prognostic implications for patients eligible for IAT irrespective of recanalization success. Significant need exists for prospective trials evaluating which patients may benefit from blood pressure augmentation prior to treatment. Based on the available trial data as well as observational studies demonstrating worse outcomes and less successful recanalization with higher BP, it is reasonable to lower the BP in the acute setting to less than 185/110 mmHg, particularly if patients may also be candidates for IV t-PA therapy.

Another important aspect to consider when evaluating AIS patients in the initial setting is intravascular volume status. In several observational studies, up to half of patients presenting with AIS are dehydrated (defined as an elevated BUN to creatinine ratio) and this has been correlated with increased in-hospital mortality and placement in institutional care at discharge (60, 61). Patients particularly at risk for dehydration include the elderly and women, who presumably have lower muscle mass, and those prescribed diuretics prior to presenting with stroke (60, 62). In AIS, hypovolemia may impair CBF to susceptible regions of ischemia and negatively influence collateral vessel development (63). While a 2015 Cochrane Review found no difference between colloid or crystalloid parenteral fluid regimens on outcome in AIS nor any data to guide the proper volume or duration of therapy, the studies were not designed to assess for treatment benefit in the acute setting or for patients who possessed serologic markers of dehydration (64). Further studies are warranted to evaluate the impact of fluid resuscitation regimens in patients presenting with AIS and dehydration. In the meantime it is reasonable to ensure patients are not volume depleted, particularly if there has been prolonged downtime, and treat with parenteral fluids as needed to target euvolemia.

### Phase II: During Revascularization

For patients undergoing IAT, predictors of poor outcome include higher National Institutes of Health Stroke Scale (NIHSS) score, lower Alberta Stroke Program Early CT Score (ASPECTS), carotid terminus occlusion, and failed recanalization (65, 66). As in other aspects of AIS, elevated BP during IAT has been associated with worse outcomes with one large observational study identifying maximal intraprocedural SBP as the strongest hemodynamic predictor (66). In this study, patients who had favorable outcomes had an average maximal SBP of 164 mmHg compared to patients with unfavorable outcomes, who had an average maximal SBP of 181 mmHg. These findings highlight the vulnerable state that exists within reperfused tissue due to impaired autoregulation and BBB disruption with increases in systemic pressure contributing to secondary injury. However, caution must be applied before aggressively lowering elevated BP as intraprocedural drops in BP are strongly correlated with less favorable outcomes. In one study, drops in MAP of greater than 40% were identified as a particular risk factor for persistent neurologic deficits (66, 67). Given the apparent detrimental effects of either extreme of intraprocedural BP, the Society of Neuroscience in Anesthesiology and Critical Care recommends SBP be maintained between 140 and 180 mmHg during IAT with careful investigation of any episodes of hypotension that may point toward end-organ injury, bleeding or volume depletion (68).

With the emergence of IAT as a prominent treatment for patients presenting with LVO, multiple questions have arisen regarding optimal treatment practices including whether general anesthesia (GA) or conscious sedation (CS) is preferable for patients undergoing thrombectomy. CS offers the advantages of allowing for neurologic assessment throughout the procedure and eliminating delays associated with anesthesia induction. GA on the other hand may reduce the risk of aspiration and eliminates patient movement thereby potentially making the procedure safer and more technically feasible (68). Early observational studies found a strong association between GA and increased time to intervention, procedural complications, ICU length of stay, and rates of tracheostomy (69–71). Furthermore, in the North American SOLITAIRE Registry, treatment with CS was associated with a 40% higher probability of good clinical outcome and a three-fold lower risk of death compared with GA (70).

While these studies seem to provide compelling evidence that CS is preferred over GA, several important methodologic issues must be addressed. First, these were all retrospective studies and possess significant selection bias as patients treated with GA were more likely to have higher NIHSS scores, lower ASPECTS, and were more likely to have carotid terminus and vertebrobasilar occlusions as well as premorbid coronary heart disease (69, 70, 72–75). In several studies, after controlling for baseline stroke severity with ASPECTS or NIHSS, the negative impact of GA was no longer statistically significant (72, 73). Second, a common observation in many of these analyses was that GA is associated with significant hypotension and fluctuations in blood pressure, particularly during the induction phase of anesthesia (70, 73, 75, 76). In a study by Davis et al., an intraprocedural SBP > 140 mmHg was associated with good outcomes however this was only achieved in 4% of patients treated with GA compared with 60% receiving CS (75). Likewise in the MR CLEAN trial, GA use was associated with larger drops in MAP with longer episodes of hypotension despite increased use of vasopressors (76). These observations highlight the impact that anesthetic agents can have on vascular tone and the potential for precipitating hypotension in AIS. However, these risks are not isolated to GA alone. In a study evaluating patients treated with CS, lower MAP prior to recanalization was found to be an independent predictor of outcome such that for every 10 mm Hg decrease below 100 mmHg there was a 28% reduced probability of favorable outcome, while a MAP drop of >10% conferred the highest risk of death or disability (77).

Recently three randomized controlled clinical trials have been published comparing the impact of CS to GA in patients treated with IAT. In contrast to prior retrospective analyses, patients treated using CS did not have better outcomes compared to those who underwent GA and in both the Sedation vs. Intubation for Endovascular Stroke Treatment (SIESTA) and General or Local Anesthesia in Intra-arterial Therapy (GOLIATH) trials, GA was associated with higher rates of functional independence at 3-months (78–80). While the time to groin puncture was slightly prolonged for GA, the overall time to reperfusion was significantly decreased and rates of successful recanalization were improved in the GOLIATH study, highlighting the potential benefit of GA in improving procedural success (78). Another key insight from these trials is the importance of aggressive intraprocedural BP control with regard to patient outcomes. While GA was associated with more frequent drops in MAP > 20% from baseline compared to CS, there was no significant difference in large falls in MAP (either defined as >40% decline or MAP < 70 mmHg) between GA and CS (78, 80). The striking discrepancies of these findings compared to prior observational studies may be related to increased vigilance over intraprocedural hypotension and strict protocols to maintain a narrow SBP goal of 140–160 mmHg and MAP > 70 mmHg (78, 79). These trials provide overwhelming evidence that both CS and GA are reasonable approaches in the management of patients undergoing IAT so long as there is strict control of BP and systems in place to ensure minimal delays are encountered while preparing patients for the interventional suite and throughout the recanalization procedure. Furthermore, in cases where potential contraindications for CS exist it may be more advantageous to start with GA rather than converting the type of anesthesia emergently in a less controlled environment. This is emphasized in the GOLIATH trial where four patients initially assigned to CS had to be converted to GA due to movement or loss of airway protection and ultimately sustained extensive infarcts (78).

### Phase III: Following Revascularization

The AHA ischemic stroke guidelines recommend maintaining a BP < 180/105 mmHg for at least 24 h in patients treated with either IV t-PA or IAT to promote perfusion to ischemic territories while mitigating potential risks of intracranial hemorrhage (16, 53). In patients treated with IV t-PA alone for LVO, these recommendations make sense from a pathophysiologic standpoint as individual recanalization status is often unknown in the clinical setting and in studies where angiography was performed the rates of early revascularization are only around 20% (81, 82). Conversely, IAT is associated with recanalization in 70–80% of cases and this can be readily confirmed during the procedure by complete anterograde reperfusion or reperfusion in more than half of the previously occluded territory (Thrombolysis in Cerebral Infarction [TICI] scores of 3 and 2b respectively) (83). In these patients, the risk of reperfusion injury with pressures approaching 180/105 mmHg could conceivably exceed that of hypoperfusion with lower BP targets.

After successful recanalization, there is often a significant spontaneous decline in BP over 12–24 h compared to patients with persistent occlusion (84, 85). For both recanalized and non-recanalized patients, sustained elevations in BP over the first 24–48 h after treatment have been identified as a risk factor for intracranial hemorrhage as well as worse functional outcomes (86–89). Similarly, blood pressure variability is more common in patients with poor or incomplete recanalization and has been correlated with larger infarct size, intracranial hemorrhage, and worse outcomes following thrombectomy (85, 89–91). Since the majority of these studies are observational, questions remain regarding the causality of these relationships. For example, work done by Delgado-Mederos and colleagues found that blood pressure variability was associated with increases in DWI lesion growth, though this relationship was only present in patients with absent recanalization, calling into question whether blood pressure variability itself is a risk factor for cerebral injury or a marker of more severe stroke (85). Despite these limitations, several interesting observations have been made when comparing patients based on recanalization status. In a Portuguese study of patients following thrombolysis, individuals with poor recanalization exhibited a U-shaped relationship of BP and outcomes with a nadir of 120–130 mmHg, similar to other reports in AIS, while those with successful revascularization demonstrated a linear relationship with more favorable outcomes occurring at the lowest pressures (<110 mmHg) (88). These results indicate that the balance between hypoperfusion and reperfusion injury may be shifted in patients following good recanalization such that the risks of exacerbating ischemia is lessened under physiologic parameters while higher systemic pressures directly contribute to cerebral injury. Similar findings were reported in a large cohort of patients following IAT where the mean SBP for intracranial hemorrhage was lower following successful recanalization (170 vs. 196 mmHg) indicating a difference in thresholds for reperfusion injury depending on the degree of vessel recanalization (92).

Though randomized trials of BP control following IAT are lacking, Goyal et al. published a single center's experience with different BP targets following IAT over 4 years. Following good reperfusion, patients were either assigned to permissive (<220/110 mmHg or 180/110 mmHg if IV t-PA also administered), moderate (<160/90 mmHg) or intensive (<140/90 mmHg) BP targets in a non-randomized fashion (87). While patients in the moderate and intensive groups received antihypertensive agents more frequently, the 3-month mortality rate in these groups was significantly lower (6.5%) compared to those with a permissive threshold (28.7%). Due to the non-controlled retrospective study design, confounding between BP targets and cohort populations cannot be excluded and as such the results must be viewed with care. Regardless, this study provides evidence that a lower BP threshold may be beneficial in patients who have achieved good recanalization status. With the establishment of IAT as the standard of care for LVO, prospective multicenter studies are now warranted to better evaluate optimal BP targets in patients with complete, partial or absent recanalization status following attempted thrombectomy. In the meantime, it may be reasonable to target a lower BP goal for patients with excellent reperfusion (TICI 2b/3) and minimal infarct volume, as was the case in the DAWN trial where patients were assigned to an intensive (<140 mmHg) goal in order to prevent reperfusion injury (93). In cases where there is incomplete or poor reperfusion, substantially less data exists to help guide management. However, a higher BP target is reasonable in an attempt to prevent further ischemia particularly if there is evidence of a fluctuating neurological examination in the context of changes in systemic BP.

## Pharmacology

Various agents are routinely used to manage hypertension in stroke however clinical trials utilizing BP agents in AIS have been inconclusive with some reports of marginal or no benefit while others suggest a risk of more severe disability in treated patients. Studies using angiotensin-converting enzyme (ACE) inhibitors or calcium channel blockers within the acute hypertensive phase of AIS have demonstrated significant acute reductions in blood pressure however this was not accompanied by changes in death or disability at 14 or 90 days (94–96). Moreover in the beta blocker stroke trial (BEST), the use of either atenolol or propranolol immediately following AIS was associated with a trend toward increased mortality, particularly among elderly individuals (97). In keeping with these disparate findings a 2014 Cochrane Review of 26 trials found insufficient evidence to support immediate resumption or routine administration of BP agents during the acute phase of stroke (98). It is important to note that many of these trials included participants with primary intracerebral hemorrhage and none were specific to patients treated either IV t-PA or IAT, further underscoring the need for trials specifically evaluating BP targets following revascularization therapy. One trial which may aid in bridging this gap in knowledge is the ENhanced Control of Hypertension ANd Thrombolysis strokE stuDy (ENCHANTED) which is a 2 × 2 randomized controlled trial with one arm evaluating the impact of early intensive BP lowering to a SBP of 130–140 mmHg in patients who are eligible for thrombolytic therapy (99). Given the current clinical equipoise of different agents on outcomes, the selection is often driven by therapy-specific adverse reactions and availability. In general, agents with a fast onset of action and short duration are preferable in the acute setting to rapidly achieve hemodynamic goals and avoid prolonged periods of hypotension. Table 2 summarizes the pharmacology of several of the most commonly used agents for acute blood pressure management.

**Table 2:** Intravenous antihypertensives for AIS.

**Drug**	**Dosing**	**Administration**	**Onset of action (min)**	**Duration**	**Clinical pearls**
Labetalol	10–20 mg IV over 1-2 min	IV bolus, infusion	2–5	2–4 h	Bradycardia, contraindicated in >1st degree heart block and cardiogenic shock
Hydralazine	10–20 mg IV, repeat every 4–6 h PRN maximum 40 mg	IV bolus	10–20	Up to 12 h	Tachycardia, drug-induced lupus erythematosus, increased intracranial pressure
Enalaprilat	0.625–1.25 mg IV every 6 h	IV bolus	<15	Up to 6 h	Contraindicated in patients with history of angioedema related to an ACE inhibitor, caution in bilateral renal artery stenosis, caution in hypovolemia
Nicardipine	5 mg/h IV, uptitrate 2.5 mg/h every 5–15 min, maximum 15 mg/h	IV infusion	5–15	4–6 h	Contraindicated in advanced aortic stenosis
Clevidipine	1–2 mg/h IV, titrate by doubling the dose every 2–5 min until desired BP reached; maximum 21 mg/h	IV infusion	2–4	5–15 min	Hypertriglyceridemia, contains soy, avoid in patients with defective lipid metabolism, limited data with use > 72 h
Sodium Nitroprusside	0.3–0.5 mcg/kg/min IV (best to avoid doses above 2 mcg/kg/min)	IV infusion	1–2	2–3 min	Cyanide toxicity, increased intracranial pressure
Glyceryl Trinitrate	5 mg/day	Transdermal	30–60	Duration of application, typically 12–14 h	Contraindicated with phosphodiesterase-5 inhibitor, tachyphylaxis, possible increase in intracranial pressure
Urapidil	10–50 mg IV followed by 4–8 mg/h	IV bolus, infusion (also available oral for maintenance therapy)	2–5	Up to 4 h	Nausea, dizziness, headaches. Contraindicated in aortic isthmus stenosis or arteriovenous shunt

### Labetalol

Labetalol is a mixed α and β adrenergic blocker often used to control blood pressure post-AIS. Though it has less β-1 activity compared to other beta blockers, labetalol can exert negative chronotropic effects thereby limiting its utility in patients with significant bradycardia (100). However, it has minimal impact on either CBF or oxygen consumption making it a suitable agent for patients with AIS or other intracranial pathology (101).

### Nicardipine

As a dihydropyridine calcium channel blocker, nicardipine is more selective for vascular rather than myocardial calcium channels and generally exerts a neutral effect on heart rate (102). When compared to labetalol, nicardipine allows for faster and more controlled reduction in BP with significantly less variability (100, 101). Despite these apparent advantages, head-to-head studies have found no differences in clinical outcomes between the two agents and the cost associated with nicardipine therapy is substantially higher (101, 103).

### Clevidipine

Clevidipine is a newer dihydropyridine calcium channel blocker which is also included as a treatment modality in the AHA 2018 guidelines (16). Similar to nicardipine, clevidipine generally does not decrease heart rate and in retrospective analysis has similar efficacy in lowering BP though requires less volume to be administered which may be optimal in patients with volume overload (104, 105). Since clevidipine is formulated in a lipid emulsion, there is an inherent risk of hypertriglyceridemia and pancreatitis for which there is a recommended a daily maximum of 1,000 mL (or about 21 mg/h per day) (106).

### Hydralazine

Hydralazine is a direct acting vasodilator that is often used for hypertensive emergencies (107). Despite its effectiveness in lowering BP, hydralazine can increase intracranial pressure (ICP) while simultaneously lowering MAP, leading to decreased perfusion pressure and increasing the risk of ischemia (108). Additionally, hydralazine has a prolonged and often unpredictable effect on BP which can contribute to precipitous drops in pressure and significant variability (109). For these reasons, hydralazine is less preferred in AIS however can be considered when other agents are not available.

### Enalaprilat

Enalaprilat, the active metabolite of enalapril, is an intravenous angiotensin-converting enzyme (ACE) inhibitor which can lower BP without impacting cardiac chronotropy (107). Additionally, ACE inhibitors are thought to be neutral with respect to ICP making enalapril potentially beneficial in patients with intracranial pathology (110). A limitation however is the long duration of action, 12–24 h, which limits the ability to titrate the agent to specific BP goals (107). Furthermore, caution must be applied when using ACE inhibitors in the setting of tPA administration as there is an increased risk of orolingual angioedema which is uncommon though can be life-threatening (111).

### Sodium Nitroprusside

Sodium nitroprusside is a potent venous and arterial vasodilator often used as an infusion in hypertensive emergency (112). However, due to its vasodilatory effects on cerebral vasculature, sodium nitroprusside can lead to increases in ICP in patients with impaired autoregulation by increasing the volume of blood within the intracranial vault (113, 114). Additionally, the sodium nitroprusside compound contains cyanide which can accumulate leading to toxicity (112). Patients at risk for this include those administered moderate to high doses as well as those with hypoalbuminemia or undergoing cardiopulmonary bypass (115). In summary, despite the potent anti-hypertensive properties of nitroprusside, the potential for impacting cerebral blood volume and ICP make it less ideal in AIS and other forms of intracranial pathology. However, it can be considered in cases where other agents are not available or are contraindicated due to patient-specific characteristics.

### Glyceryl Trinitrate

Glyceryl trinitrate, or nitroglycerin, is a nitric oxide donor that primarily causes venodilation, as well as arterial dilation at high doses, thereby effectively reducing preload and BP (106). Glyceryl trinitrate is most commonly used for acute myocardial infarction and unstable angina due to its ability to reduce cardiac oxygen demand, but there is recent literature evaluating its use in AIS. In the ENOS (Efficacy of Nitric Oxide in Stroke) Trial, which primarily took place in Europe and Asia, the investigators studied the use of transdermal glyceryl trinitrate patch in AIS or hemorrhagic stroke patients at a dose of 5 mg daily for 7 days compared to placebo. While transdermal glyceryl trinitrate was effective in lowering blood pressure, there was no significant improvement in functional outcome at 90 days (116). Of note, there is literature from primarily small observational studies that suggests nitroglycerin may increase ICP, although the clinical significance of this is uncertain (117).

### Urapidil

Urapidil is an antihypertensive agent available throughout Europe and Asia and recommended by the European Stroke Initiative (EUSI) though it is not currently approved by the United States Food and Drug Association (FDA). Urapidil is a unique antihypertensive agent which exerts peripheral vasodilation through alpha-1-adrenoreceptor antagonism as well as sympatholytic effects via serotonin 5HT1A receptor stimulation (118). Animal studies have also demonstrated potential neuroprotective effects of urapidil thereby further increasing its potential for treating patients with AIS (119). While urapidil has generally be considered to have neutral effects on ICP, more recent studies including two patients with head injury as well as a cohort of normal volunteers suggest that administration of the agent may lead to increases in ICP (119, 120).

### Induced Hypertension

In animal models of AIS, induced hypertension (IH) improves CBF to the ischemic territory and reduces final infarct volume compared to normotensive controls (121, 122). Phenylephrine is often utilized in this setting due to its pure α-1 receptor agonist properties which causes peripheral vasoconstriction without significantly impacting the cerebral vasculature thereby improving perfusion pressure (123). Though IH is sometimes used in clinical practice, particularly if there is evidence of changes in the neurologic exam across different blood pressures, little clinical data is available to substantiate its use. In a 2001 pilot study using phenylephrine in patients with AIS, targeting a BP of at least 160 mmHg or a 20% increase relative to admission was associated with short-term improvement in the NIHSS in over half of patients without any associated complications (124). A subsequent pilot clinical trial using IH in patients with large diffusion-perfusion mismatch demonstrated a significant improvement in NIHSS, cognitive scores, and hypoperfused tissue over 3 days compared to controls (125). In light of these findings, further studies are warranted to determine the clinical utility of IH. This is the focus of the randomized multicenter SETIN-HYPERTENSION phase III trial (NCT01600235) which aims to determine the safety and efficacy of phenylephrine in patients with non-cardioembolic stroke.

## Conclusion

Hemodynamic management in AIS is an involved and complex process that aims to balance the competing interests of supporting CBF to the ischemic penumbra while avoiding reperfusion injury. Decades of observational studies have demonstrated a U-shaped association between BP and stroke outcomes. However, studies aimed at controlling hemodynamics have been inconclusive. In the setting of thrombolytic therapy, the pendulum may shift from a higher risk of hypoperfusion to that of reperfusion injury, therefore making BP control particularly paramount. This is especially relevant in IAT where rates of LVO recanalization are substantially higher than in other treatment modalities and revascularized patients could benefit from lower BP thresholds to prevent intracranial hemorrhage and cerebral edema. Well-designed prospective multicenter controlled clinical trials are now warranted to better understand the relationship between various BP goals during and post-embolectomy with particular focus on interactions with recanalization status. In the meantime, careful monitoring and management of hemodynamics is essential for prevention of significant hypo- or hypertension as well as minimizing BP variability in order to best promote tissue recovery while preventing secondary injury.

## Author Contributions

JH and JV manuscript concept and design. JV initial manuscript draft. MT and JH critical review of manuscript and addition of sections. All authors reviewed, edited, and approved the final version.

### Conflict of Interest Statement

The authors declare that the research was conducted in the absence of any commercial or financial relationships that could be construed as a potential conflict of interest.
